# Sp7 Transgenic Mice with a Markedly Impaired Lacunocanalicular Network Induced Sost and Reduced Bone Mass by Unloading

**DOI:** 10.3390/ijms23063173

**Published:** 2022-03-15

**Authors:** Takeshi Moriishi, Takuro Ito, Ryo Fukuyama, Xin Qin, Hisato Komori, Hitomi Kaneko, Yuki Matsuo, Noriaki Yoshida, Toshihisa Komori

**Affiliations:** 1 Department of Cell Biology, Nagasaki University Graduate School of Biomedical Sciences, Nagasaki 852-8588, Japan; moriishi@nagasaki-u.ac.jp; 2 Basic and Translational Research Center for Hard Tissue Disease, Nagasaki University Graduate School of Biomedical Sciences, Nagasaki 852-8588, Japan; bb55312207@ms.nagasaki-u.ac.jp (T.I.); qinxin_19861009@163.com (X.Q.); hkomori@nagasaki-u.ac.jp (H.K.); kaneko-h-1990@nagasaki-u.ac.jp (H.K.); ysmatsuo@nagasaki-u.ac.jp (Y.M.); 3 Department of Orthodontics and Dentofacial Orthopedics, Nagasaki University Graduate School of Biomedical Sciences, Nagasaki 852-8588, Japan; nori@nagasaki-u.ac.jp; 4 Laboratory of Pharmacology, Hiroshima International University, Kure 737-0112, Japan; r-fukuya@hirokoku-u.ac.jp; 5 Japan Society for the Promotion of Science International Research Fellow, Tokyo 102-0083, Japan

**Keywords:** osteocyte, Sp7, Sost, unloading, mechanical stress, canaliculus, lacunocanalicular network, apoptosis, bone formation, bone resorption

## Abstract

The relationship of lacunocanalicular network structure and mechanoresponse has not been well studied. The lacunocanalicular structures differed in the compression and tension sides, in the regions, and in genders in wild-type femoral cortical bone. The overexpression of Sp7 in osteoblasts resulted in thin and porous cortical bone with increased osteoclasts and apoptotic osteocytes, and the number of canaliculi was half of that in the wild-type mice, leading to a markedly impaired lacunocanalicular network. To investigate the response to unloading, we performed tail suspension. Unloading reduced trabecular and cortical bone in the Sp7 transgenic mice due to reduced bone formation. Sost-positive osteocytes increased by unloading on the compression side, but not on the tension side of cortical bone in the wild-type femurs. However, these differential responses were lost in the Sp7 transgenic femurs. Serum Sost increased in the Sp7 transgenic mice, but not in the wild-type mice. Unloading reduced the Col1a1 and Bglap/Bglap2 expression in the Sp7 transgenic mice but not the wild-type mice. Thus, Sp7 transgenic mice with the impaired lacunocanalicular network induced Sost expression by unloading but lost the differential regulation in the compression and tension sides, and the mice failed to restore bone formation during unloading, implicating the relationship of lacunocanalicular network structure and the regulation of bone formation in mechanoresponse.

## 1. Introduction

Osteoblast differentiation is regulated by transcription factors, including Runx2, Sp7, and Dlx5, and many signaling pathways, including Wnt, hedgehog, Fgf, and Pthlh [1]. Runx2 increases the proliferation of osteoblast progenitors and induces their commitment into osteoblast lineage cells through the reciprocal regulation with signaling pathways, including hedgehog, Fgf, Wnt, and Pthlh. Sp7 is a zinc finger-containing transcription factor, which belongs to the Sp/KLH (Kruppel-like Factor) family and is essential for osteoblast differentiation [2]. Runx2 induces Sp7 expression in preosteoblasts, and both are required for further differentiation into osteoblasts and major bone matrix protein gene expression in the osteoblasts [1,3,4]. Osteoblasts are then embedded into the bone matrix and become osteocytes.

Osteocytes establish an extensive intracellular communication system through their abundant processes, which are connected with the processes of neighboring osteocytes and/or osteoblasts on the bone surface via gap junctions throughout the bone. Furthermore, osteocytes establish an extracellular communication system via canaliculi, which enables fluid flow and nutrient transport by connecting with neighboring osteocytes, blood vessels, bone surface cells, and bone marrow [5]. The osteocyte network is widely considered to be a mechanosensory and mechanotransduction system [6,7,8,9,10,11,12]. Sheer stress on osteocytes that is induced by fluid flow through the proteoglycan matrix in the lacunocanalicular network has been proposed as an important mechanism for mechanoresponse [13]. Moreover, shear stress decreases when the canalicular surface area is reduced [14]; the compressive strain, fluid flow velocity, and bone formation are parallelly changed in mouse tibiae [15]; the densities of lacunae and canaliculi decrease in humans and mice with age; and aging leads to the dysregulation of the mechanoresponse [16,17], suggesting the importance of the lacunocanalicur structure for mechanoresponse. However, direct evidence showing the relationship of the lacunocanalicular structure and mechanoresponse is lacking due to the absence of appropriate animal models. Further, the heterogeneity of the lacunocanalicular structure within the osteons is observed in human femoral cortical bone [18], however, the differences of the lacunocanalicular structure in the compression and tension sides of the cortical bone, in the periosteal and endosteal sides of cortical bone, and in genders have not been reported in any species.

Unloaded conditions are generated by tail suspension or hind limb immobilization, and the bone volume is reduced by inhibited bone formation and enhanced bone resorption in tail suspension using C57BL/6 mice, which are sensitive to unloading [19]. Further, Sost-positive osteocytes are increased in the compression side of the tibia, and Rankl expression is upregulated in osteoblasts by tail suspension [12]. The overexpression of Bcl2 in osteoblasts results in massive osteocyte apoptosis, the number of osteocyte processes is markedly reduced, and the mice do not respond to unloading. Although mechanical stress alters Sost expression [20], the mice do not induce Sost expression in unloading [12]. These findings suggest that the osteocyte network is required for the response to unloading and the Sost induction in unloading. As the lacunocanalicular network is completely disrupted in Bcl2 transgenic (tg) mice, the mice could be a model for examining the relationship of the lacunocanalicular structure and mechanoresponse. However, the complete disruption of the lacunocanalicular network due to the massive osteocyte apoptosis makes the evaluation difficult. Therefore, an appropriate mouse model for examining the requirement of an organized lacunocanalicular structure in the mechanoresponse is still lacking, and the relationship of the lacunocanalicular network structure and the mechanoresponse remains to be studied.

We previously reported that the overexpression of Runx2 or Sp7 in osteoblasts using the 2.3-kb Col1a1 promoter inhibits osteoblast maturation [21,22]. Furthermore, the overexpression of Runx2 markedly reduced the number of osteocytes, whereas that of Sp7 reduced the number of osteocyte processes, leading to a markedly impaired lacunocanalicular network [21,22]. We report the lacunocanalicular network in Sp7 tg mice in detail and how Sp7 tg mice with a markedly impaired lacunocanalicular network respond to unloading, showing the relationship of the lacunocanalicular network structure and the regulation of bone formation and Sost expression in mechanoresponse.

## 2. Results

### 2.1. Cortical Bone Was Thin and Porous in Sp7 tg Mice, but the Trabecular Bone Volume Was Similar to That in Wild-Type Mice in Micro-CT Analysis

**Figure 1:**
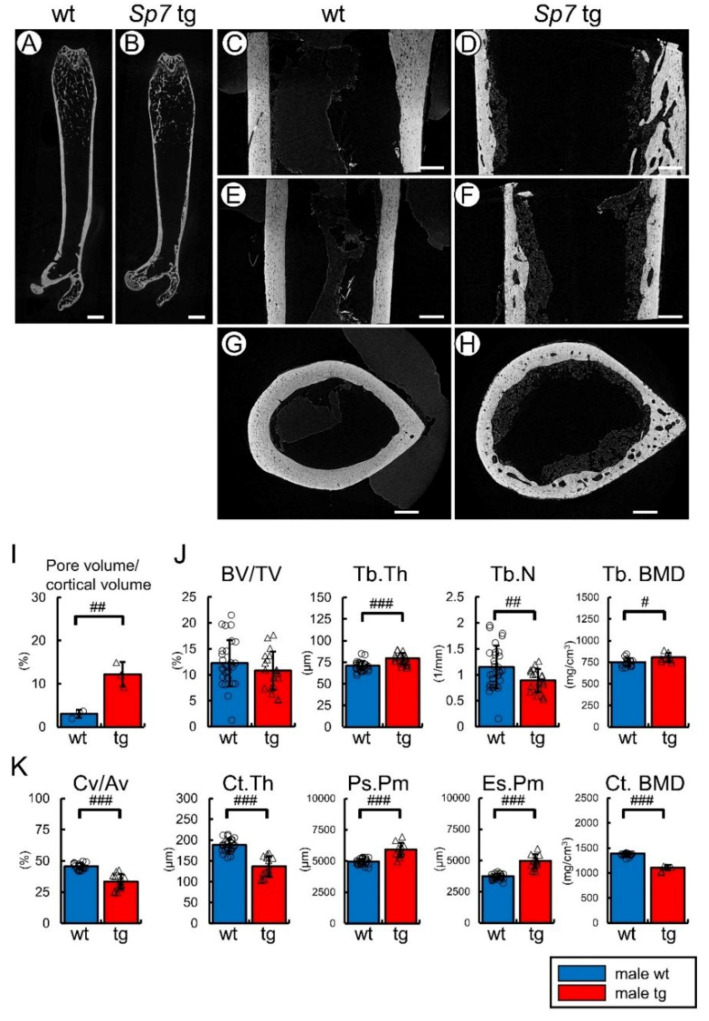
Micro-CT analysis of femurs: (**A**–**I**) Analysis of the femurs by high resolution micro-CT. (**A**,**B**) Images of the frontal sections of the whole femur in the male wild-type (**A**) and Sp7 tg (**B**) mice. (**C**–**H**) Images of the frontal section (**C**,**D**), sagittal section (**E**,**F**), and cross-section (**G**,**H**) of the shaft of femurs in the wild-type (**C**,**E,G**) and Sp7 tg (**D**,**F**,**H**) mice. Scale bars: 1 mm (**A**,**B**); 200 μm (**C**–**H**). (**I**) Pore volume/cortical volume. A total of three mice in each genotype were analyzed. (**J**,**K**) Quantitative analysis of trabecular (**J**) and cortical (**K**) bone of the male wild-type and Sp7 tg mice by micro-CT. (**J**) The trabecular bone volume (bone volume/tissue volume, BV/TV), trabecular thickness (Tb.Th), trabecular number (Tb.N), and trabecular bone mineral density (Tb.BMD). (**K**) The cortical bone ratio (cortical bone volume/all bone volume, Cv/Av), cortical thickness (Ct.Th), periosteal perimeter (Ps.Pm), endosteal perimeter (Es.Pm), and cortical bone mineral density (Ct.BMD). The number of mice that were analyzed; wild-type: 30 (BMD: 16), tg: 19 (BMD: 8). # *p* < 0.05, ## *p* < 0.01, ### *p* < 0.001 by Student’s *t*-test.

The structure of the femoral cortical bone in the male Sp7 tg mice under the control of the 2.3-kb Col1a1 promoter was first examined by micro-CT with high resolution. The cortical bone of the Sp7 tg mice had many holes, which were few in the wild-type bone (Figure 1A–H). The pore volume in the Sp7 tg cortical bone was higher than that in the wild-type cortical bone (Figure 1I). Next, the trabecular and cortical bone of femurs was quantitatively compared between the wild-type and Sp7 tg male mice by micro-CT (Figure 1J,K). Although the bone volume of the trabecular bone was similar between the wild-type and Sp7 tg mice, the trabecular thickness and bone mineral density (BMD) were increased but the trabecular number was reduced in the Sp7 tg mice compared with those in the wild-type mice (Figure 1J). The cortical bone ratio and the cortical thickness were lower, the periosteal and endosteal perimeters were larger, and the cortical BMD was lower in the Sp7 tg mice than those in the wild-type mice (Figure 1K). Thus, the Sp7 tg mice had thin and porous cortical bone but the trabecular bone was grossly similar to that in the wild-type mice.

### 2.2. TRAP-Positive Cells and TUNEL-Positive Osteocytes Were Increased in Cortical Bone of Sp7 Tg Mice of Both Sexes

**Figure 2:**
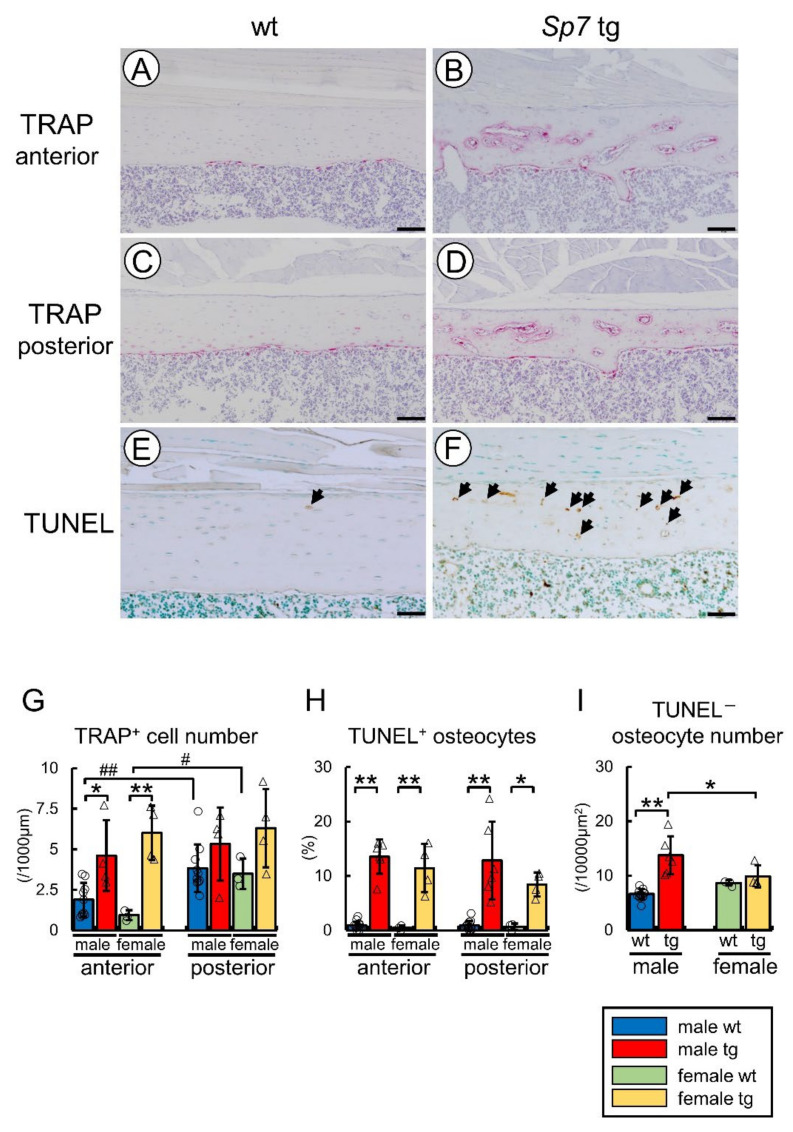
TRAP and TUNEL staining: (**A**–**F**) TRAP staining in the anterior cortical bone (**A**,**B**) and the posterior cortical bone (**C**,**D**) and TUNEL (**E**,**F**) staining in the anterior cortical bone in the femurs of female wild-type (**A**,**C**,**E**) and Sp7 tg (**B**,**D**,**F**) mice. Arrows in E and F show TUNEL-positive lacunae. Scale bars: 200 μm (**A**–**D**); 100 μm (**E**,**F**). (**G**) The number of TRAP-positive cells in the anterior and posterior cortical bone. The number of mice that were analyzed; male wt: 10, male tg: 4, female wt: 3, female tg: 4. (**H**) Frequencies of TUNEL-positive lacunae in the anterior and posterior cortical bone. (**I**) The number of TUNEL-negative osteocytes. The number of mice that were analyzed in (**H**,**I**); male wt: 13, male tg: 6, female wt: 3, female tg: 4. *^,^# *p* < 0.05. **^,^## *p* < 0.01 by Tukey–Kramer test * and Student’s *t*-test #.

The femurs of the Sp7 tg mice were compared with those of the wild-type mice by histological analysis. The hematopoietic cells and tartrate-resistant acid phosphatase (TRAP)-positive osteoclasts were observed inside the cortical bone of the Sp7 tg mice (Figure 2B,D). The numbers of TRAP-positive cells in the anterior cortical bone but not the posterior cortical bone of the Sp7 tg mice were higher than those of the wild-type mice in both males and females (Figure 2G). The number of TRAP-positive cells in the posterior cortical bone was higher than that in the anterior cortical bone in the wild-type mice in both males and females due to the higher number of TRAP-positive cells in the endosteum, which resulted in a lack of the difference of TRAP-positive cell number between the wild-type and Sp7 tg mice in the posterior cortical bone (Figure 2A,C,G). In contrast, the TRAP-positive cells were similarly observed inside the cortical bone and in the endosteum between the anterior and posterior cortical bone in the Sp7 tg mice in both males and females (Figure 2B,D,G). To search a possible reason for the increase of TRAP-positive cells in the cortical bone, terminal deoxynucleotidyl transferase-mediated dUTP nick end labeling (TUNEL) staining was performed using femoral sections because osteocyte apoptosis induces bone resorption [23,24,25]. The frequency of TUNEL-positive osteocytes in the Sp7 tg mice was markedly increased compared with that in the wild-type mice in the anterior and posterior cortical bone in both males and females (Figure 2E,F,H). Although the increase of TRAP-positive cells was more prominent in the female Sp7 tg mice than the male Sp7 tg mice in the anterior cortical bone, the frequency of TUNEL-positive osteocytes was similar between the male and female Sp7 tg mice. The number of TUNEL-negative osteocytes in the male but not the female Sp7 tg mice was increased as compared with that in the wild-type mice, and the male Sp7 tg mice had more TUNEL-negative osteocytes than the female Sp7 tg mice (Figure 2I).

### 2.3. The Bone Formation Rates in Trabecular and Cortical Bone in Sp7 Tg Mice Were Similar to Those in Wild-Type Mice, but the Osteoclast Parameters Were Increased in Sp7 Tg Mice in Bone Histomorphometric Analysis

Based on bone histomorphometric analysis of the trabecular bone of femurs, the osteoid thickness and mineral apposition rate in the Sp7 tg mice were lower than those in the wild-type mice, while the osteoclast parameters, including the osteoclast surface, osteoclast number, and eroded surface, were higher in the Sp7 tg mice than those in the wild-type mice (Figure 7). The osteocyte number was increased in the Sp7 tg mice compared with the wild-type mice. The other parameters, including osteoid surface, osteoblast surface, osteoblast number, mineralizing surface, and bone formation rate, in trabecular bone, and all dynamic bone histomorphometric parameters in cortical bone were similar between the wild-type and Sp7 tg mice (Figures 7 and 8).

### 2.4. The Direction of Collagen Fibers Was Uniformly Longitudinal in Anterior Cortical Bone but Mildly Disturbed in Posterior Cortical Bone of Male Wild-Type Femurs, and the Direction Was Completely Disturbed in Anterior and Posterior Cortical Bone of Sp7 Tg Femurs

**Figure 3:**
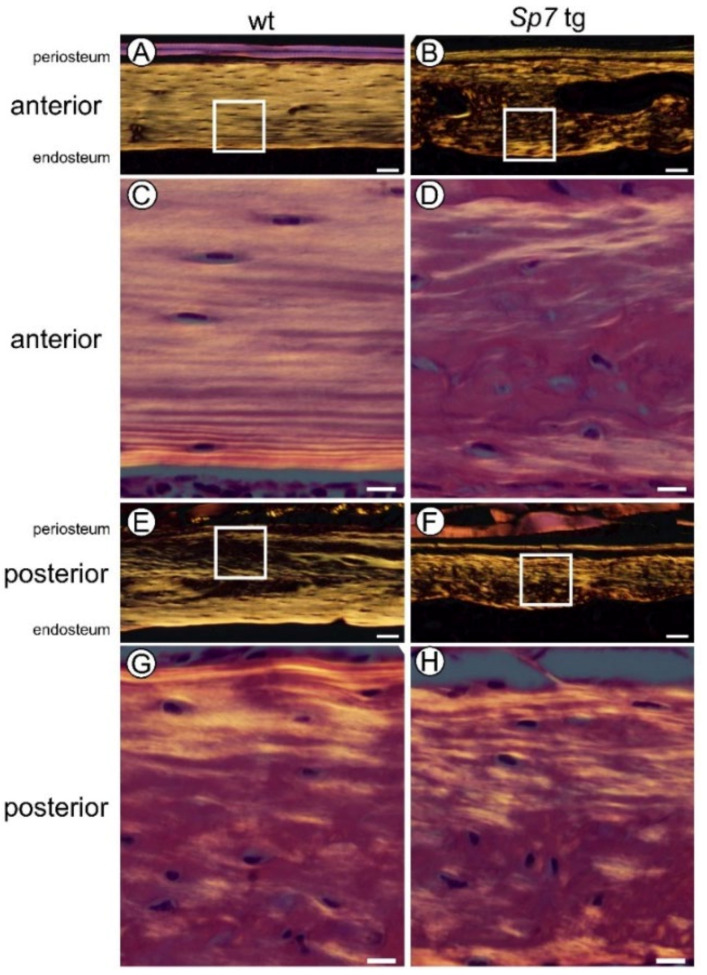
Polarized microscopy of the cortical bone at diaphyses of femurs in male wild-type and Sp7 tg mice at 14 weeks of age: Anterior (**A**,**B**) and posterior (**E**,**F**) cortical bone in the wild-type (**A**,**E**) and Sp7 tg (**B**,**F**) mice. The boxed regions in (**A**,**B**,**E**,**F**) are magnified in (**C**,**D**,**G**,**H**), respectively. Scale bars = 50 μm (**A**,**B**,**E**,**F**); 10 μm (**C**,**D**,**G**,**H**). A total of seven wild-type and three Sp7 tg mice were analyzed and the representative pictures are shown.

In mouse femurs, the anterior cortical bone is the compression side and the posterior cortical bone is the tension side. To investigate the direction of collagen fibers in the anterior (compression side) and posterior (tension side) cortical bone in males, the sections that were stained with hematoxylin and eosin (H–E) were observed by polarized microscopy. The osteocytes were spindle-shaped, and osteocytes and collagen fibers were aligned to the longitudinal direction in the anterior cortical bone, while the direction of the collagen fibers was mildly disturbed in some regions especially in the middle to the periosteum of posterior cortical bone, and the osteocytes were irregularly-orientated and rounded in the disturbed region (Figure 3A,C,E,G). In contrast, the collagen fibers ran in random directions, and the osteocytes were round and irregularly-oriented in both the anterior and posterior cortical bone in the diaphysis of the Sp7 tg femurs (Figure 3B,D,F,H).

### 2.5. The Number of Canaliculi Was Markedly Reduced and the Lacunocanalicular Network Was Severely Disorganized in Sp7 Tg Mice, and the Structure of Lacunocanalicular Network Differed in the Compression and Tension Sides, in the Periosteal and Endosteal Sides, and in Genders in Wild-Type Mice and in Sp7 tg Mice except the Lacuna Shape

**Figure 4:**
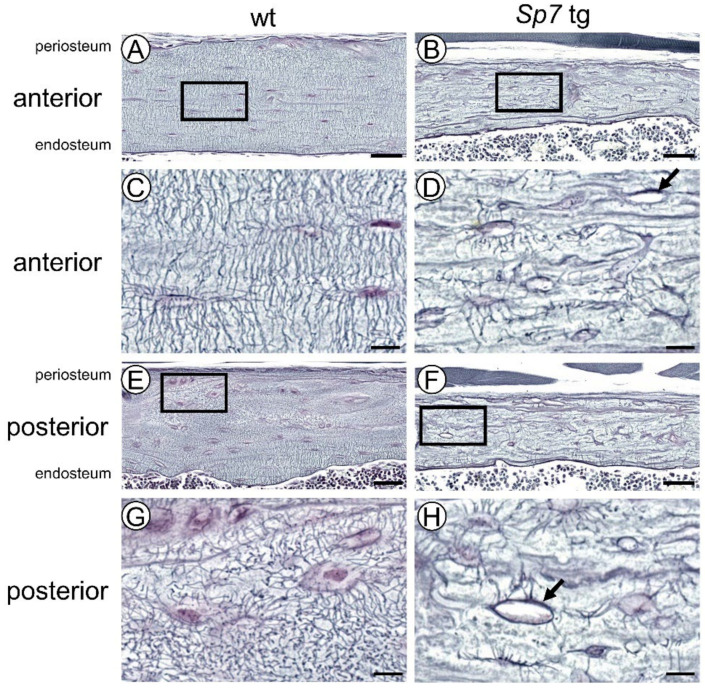
Canalicular staining of the male femoral cortical bone: Anterior (compression) (**A**,**B**) and posterior (tension) (**E**,**F**) sides of wild-type (**A**,**E**) and Sp7 tg (**B**,**F**) cortical bone. The boxed regions in (**A**,**B**,**E**,**F**) are magnified in (**C**,**D**,**G**,**H**), respectively. The arrows in (**D**,**H**) show empty lacunae. Scale bars: 50 μm (**A**,**B**,**E**,**F**); 10 μm (**C**,**D**,**G**,**H**).

To compare the bone structure in the compression and tension sides, the lacunae and canaliculi in anterior and posterior cortical bone of femurs were visualized by silver impregnation staining. In the male wild-type mice, the lacunae were spindle-shaped and aligned in the longitudinal direction of femurs, and the canaliculi were regularly aligned in the perpendicular direction to the lacunae in the diaphysis of the anterior cortical bone (Figure 4A,C). Similar to the observation by polarized microscopy, the lacunae were rounded and the alignment of the canaliculi was disorganized in some regions, especially from the middle to periosteum in the posterior cortical bone of the male wild-type femurs (Figure 4E,G). In contrast, the lacunae were round, the number of canaliculi was markedly reduced, and their orientation was disorganized in the both the anterior and posterior cortical bone in the male Sp7 tg mice (Figure 4B,D,F,H). Further, the number of stained canaliculi was extremely low around the empty lacunae (arrows in Figure 4D,H).

**Figure 5:**
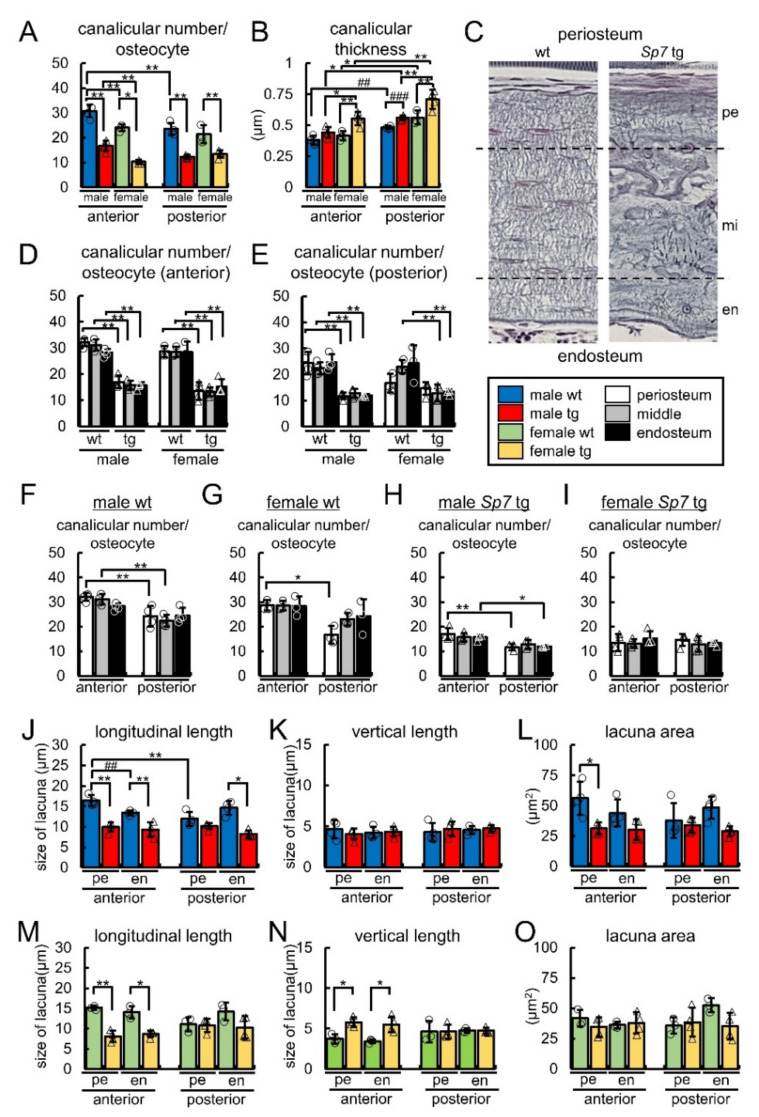
The canalicular number, canalicular thickness, and the size of the lacunae in the diaphysis of the anterior and posterior femoral cortical bone in the wild-type and Sp7 tg mice of both sexes: (**A**) The average number of canaliculi in one osteocyte. (**B**) Canalicular thickness. (**C**–**E**) The average number of canaliculi in one osteocyte in three divided regions of the anterior (**D**) and posterior (**E**) cortical bone. The number of canaliculi from lacunae with osteocytes was counted in three divided regions, one-fourth area of the periosteum side (pe), half area in the middle (mi), and one-fourth area of the endosteum side (en), as shown in (**C**), and the average number of canaliculi in one osteocyte was calculated (**D**,**E**). A total of 12 groups were compared in (**D**,**E**). (**F**–**I**) Based on the data of (**D**,**E**), the canalicular number was compared between the anterior and posterior cortical bone in the three divided regions in the male wile-type (**F**), female wild-type (**G**), male Sp7 tg (**H**), and female Sp7 tg **(I**) mice. (**J**–**O**) The longitudinal length (**J**,**M**), vertical length (**K**,**L**), and area (**L**,**O**) of lacunae in the periosteal (pe) and endosteal (en) halves of the anterior and posterior cortical bone in the male (**J**–**L**) and female (**M**–**O**) wild-type and Sp7 tg mice. The number of mice that were analyzed; male wt and tg: 4, female wt: 3, female tg: 4. * *p* < 0.05, **^,^## *p* < 0.01, ### *p* < 0.001 by Tukey-Kramer test * and Student’s *t*-test #.

The number of canaliculi from one lacuna, which includes a live osteocyte, and the canalicular thickness were measured in the anterior and posterior cortical bone in the wild-type and Sp7 tg femurs in both sexes (Figure 5A,B). The number of canaliculi in the anterior cortical bone was higher than that in the posterior cortical bone in the male wild-type mice, and that in the anterior cortical bone in males was higher than that in the females in both the wild-type and Sp7 tg mice (Figure 5A). The number of canaliculi in Sp7 tg mice was markedly reduced in both the anterior and posterior cortical bone and in both sexes compared with that in the wild-type mice (Figure 5A). The canaliculi in the posterior cortical bone were wider than those in the anterior cortical bone in the wild-type and Sp7 tg mice of both sexes (Figure 5B). Further, the canaliculi in the Sp7 tg mice were wider than those in the wild-type mice in both the anterior and posterior cortical bone in the females and the posterior cortical bone in the males, and the canaliculi in the female Sp7 tg mice were the widest in both the anterior and posterior cortical bone (Figure 5B). Thus, the compression side had more and thinner canaliculi in the males and thinner canaliculi in the females than the tension side in the wild-type mice, while the compression side had thinner canaliculi in both genders than the tension side and the males had thinner canaliculi than the females in the Sp7 tg mice. 

To further examine whether the lacunocanalicular structure differs in the locations of femoral cortical bone, the number of canaliculi in live osteocytes were analyzed in three divided regions, which are periosteal, middle, and endosteal regions, of the cortical bone as shown in Figure 5C. In each region, the canalicular number in the Sp7 tg mice was lower than that in the wild-type mice except the periosteal region of posterior cortical bone in females (Figure 5D,E). However, the canalicular number was not significantly different among the three regions in the anterior and posterior cortical bone in the wild-type and Sp7 tg mice of both sexes (Figure 5D,E). In the comparison between the anterior and posterior cortical bone in the wild-type mice, the canalicular number in the periosteal region of anterior cortical bone was higher than that of posterior cortical bone in both sexes, and the canalicular number in the middle region of the anterior cortical bone was higher than that of the posterior cortical bone in the males (Figure 5F,G). In the Sp7 tg mice, the canalicular numbers in the periosteal and endosteal regions of the anterior cortical bone were higher than those of the posterior cortical bone in males but not in females (Figure 5H,I). Thus, the differences of the canalicular number in the compression and tension sides depended on the regions, and the compression side had more canaliculi than the tension side in the periosteal region in both the wild-type and Sp7 tg mice. 

As the shape of the osteocytes was rounded in some regions especially in the middle to the periosteum in posterior cortical bone of the wild-type mice (Figure 3E,G and Figure 4E,G), the length and area of lacunae were measured in the two divided regions, which were the periosteal half and endosteal half, in the anterior and posterior cortical bone of the wild-type and Sp7 tg femurs in both sexes. In the male mice, the lacuna length in the longitudinal direction in the periosteal half of anterior cortical bone was longer than that of the posterior cortical bone in the wild-type femurs (Figure 5J). The length in the Sp7 tg femurs was shorter than that in the wild-type femurs, except the periosteal half in the posterior cortical bone (Figure 5J). The lacuna lengths in the vertical direction were similar among the eight groups (Figure 5K). The lacuna area in the periosteal half of anterior cortical bone in the wild-type femurs was larger than that in the Sp7 tg femurs, but the differences in the other comparisons were not significant (Figure 5L). In the female mice, the lacuna length in the longitudinal direction was shorter, and that in the vertical direction was longer in the Sp7 tg femurs than that in the wild-type femurs in both the periosteal and endosteal halves of the anterior cortical bone, but not in the posterior cortical bone (Figure 5M,N). There were no significant differences in the lacuna area among the eight groups in females (Figure 5O). Thus, the lacunae in the periosteal half in the compression side of the male wild-type mice was most spindle-shaped, while the shape of lacunae in Sp7 tg mice was rounded in the whole area in both genders. 

### 2.6. The Serum Levels of P1NP Were Similar, Those of Sost Were Reduced, and Osteoblast Marker Gene Expression Was Increased in Sp7 Tg Mice Compared with Those in Wild-Type Mice

We examined the serum markers for bone formation [total procollagen type 1 N-terminal propeptide (P1NP)] and bone resorption [tartrate-resistant acid phosphatase 5b (TRAP5b)]. The serum levels of the total P1NP were similar between the wild-type and Sp7 tg mice, but the serum levels of TRAP5b in the Sp7 tg mice were higher than those in the wild-type mice (Figure 9). The expressions of the osteoblast marker genes, including Runx2, Spp1, Ibsp, Col1a1, and Bglap/Bglap2, were increased in the Sp7 tg mice compared with those in the wild-type mice (Figure 10). The expressions of Rankl and Opg in the Sp7 tg mice were higher than those in the wild-type mice, and the ratio of Rankl and Opg was not significantly different between them.

In immunohistochemistry of Sost using the femoral sections, the lacunae and canaliculi were clearly stained in both the wild-type and Sp7 tg mice (Figure 11A–F). Furthermore, the staining revealed that Sost, which was produced by the osteocytes, reached the periosteum and endosteum through the canalicular network in the Sp7 tg mice with a sparse and markedly disorganized canalicular network (Figure 11B,D). However, the canaliculi that were stained with Sost were few around apoptotic or necrotic osteocytes, which were non-specifically stained with Sost, indicating that osteocyte apoptosis interrupted the lacunocanalicular network (Figure 11E,F). We compared the number of Sost-positive cells in the anterior (compression side) and posterior (tension side) cortical bone. Although Sost was expressed at similar frequencies in the anterior cortical bone between the wild-type and Sp7 tg mice, the frequency of Sost-positive cells in the posterior cortical bone of the Sp7 tg mice was lower than that of the wild-type mice (Figure 11G,H,K,L,O). Further, the serum level of Sost in the Sp7 tg mice was lower than that in the wild-type mice (Figure 11P).

### 2.7. Marked Reduction of Both Trabecular and Cortical Bone in Sp7 Tg Mice by Unloading Due to Reduced Bone Formation, and Increases of Osteoblast and Osteoclast Parameters in Wild-Type Mice but Not in Sp7 Tg Mice by Unloading

**Figure 6:**
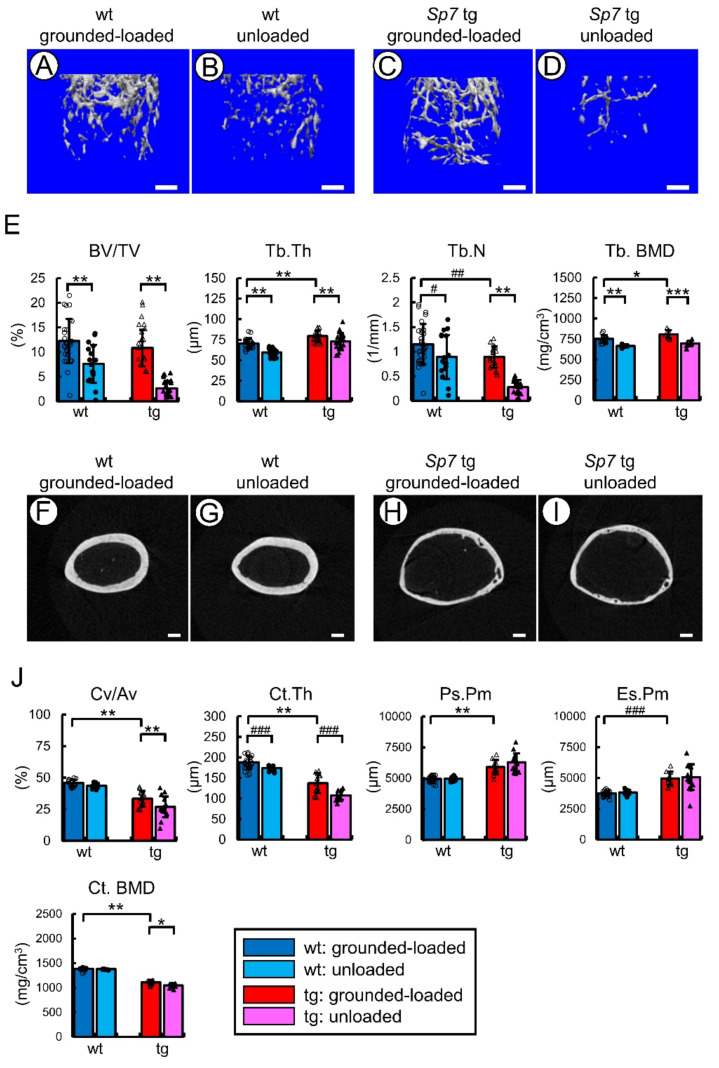
Micro-CT analysis of femurs in the grounded-loaded and unloaded mice: tail suspension was performed for two weeks using male wild-type and Sp7 tg mice. (**A**–**E**) Three-dimensional trabecular bone architecture of distal femoral metaphysis (**A**–**D**), and quantification of the trabecular bone volume (bone volume/tissue volume, BV/TV), trabecular thickness (Tb.Th), trabecular number (Tb.N), and trabecular bone mineral density (Tb.BMD) (**E**). (**F**–**J**) Micro-CT images of the cortical bone at mid-diaphysis in femurs (**F**–**I**), and the quantification of the cortical bone ratio (cortical bone volume/all bone volume, Cv/Av), cortical thickness (Ct.Th), periosteal perimeter (Ps.Pm), endosteal perimeter (Es.Pm), and cortical bone mineral density (Ct.BMD) (**J**). Scale bars: 0.5 mm (**A**–**D**); 0.2 mm (**F**–**I**). The number of mice that were analyzed; grounded-loaded wild-type: 30 (BMD: 16), unloaded wild-type: 18 (BMD: 6), grounded-loaded tg: 19 (BMD: 8), unloaded tg: 19 (BMD: 9). *^,^# *p* < 0.05, **,## *p* < 0.01, ***,### *p* < 0.001 by Tukey–Kramer test * and Student’s *t*-test #. The parameters of trabecular and cortical bone in the wild-type and Sp7 tg mice in the grounded-loaded condition in Figure 1J,K are shown for the comparison of grounded-loaded and unloaded mice in (**E**,**J**), respectively. The data were collected from two independent experiments except BMD.

**Figure 7:**
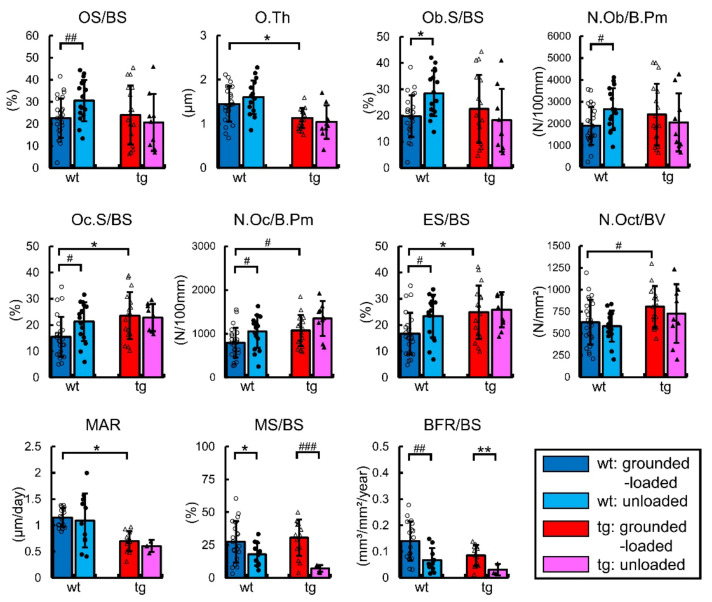
Bone histomorphometric analysis of the trabecular bone in the femurs of grounded-loaded and unloaded male mice: the osteoid surface (OS/BS), osteoid thickness (O.Th), osteoblast surface (Ob.S/BS), number of osteoblasts (N.Ob/B.Pm), osteoclast surface (Oc.S/BS), number of osteoclasts (N.Oc/B.Pm), eroded surface (ES/BS), number of osteocytes (N.Oct/BV), mineral apposition rate (MAR), mineralizing surface (MS/BS), and bone formation rate (BFR/BS) were measured in the wild-type and Sp7 tg mice. BS, bone surface; B.Pm, bone perimeter. The number of mice that were analyzed; grounded-loaded wild-type: 17–25, unloaded wild-type: 10–16, grounded-loaded tg: 13–17, unloaded tg: 3–9. *^,^# *p* < 0.05, **^,^## *p* < 0.01, ### *p* < 0.001 by Tukey–Kramer test * and Student’s *t*-test #. The data were collected from two independent experiments.

**Figure 8:**
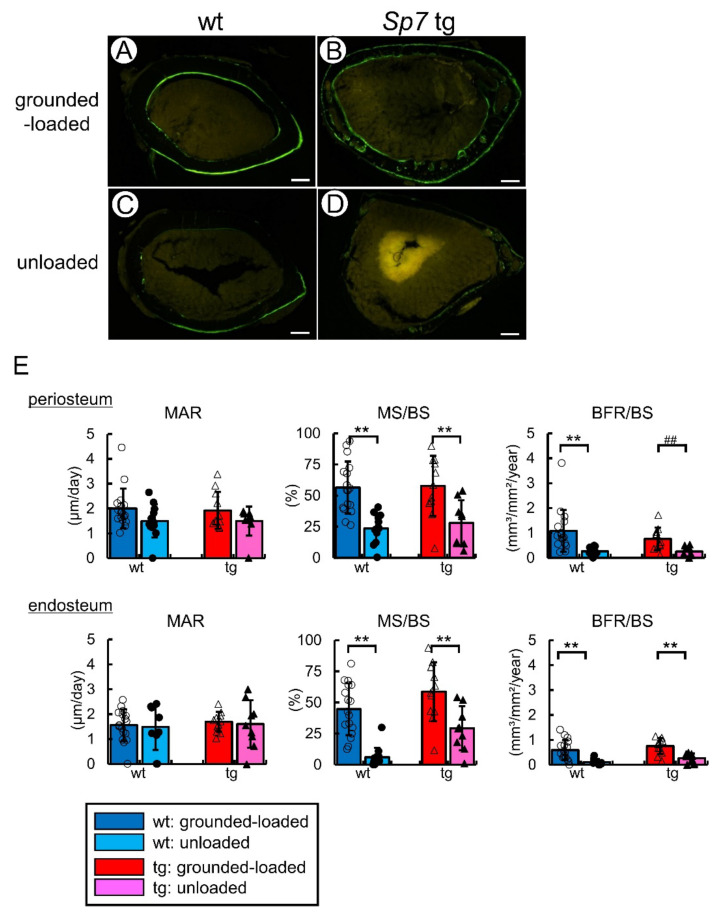
The dynamic bone histomorphometric analysis of the cortical bone in the femurs of grounded-loaded and unloaded male mice: (**A**–**D**) Cross-sections in the mid-diaphyses of femurs in the wild-type (**A**,**C**) and Sp7 tg (**B**,**D**) mice. Scale bars: 0.2 mm. (**E**) Mineral apposition rate (MAR), mineralizing surface (MS/BS), and bone formation rate (BFR/BS) in the periosteum and endosteum. The number of mice that were analyzed; grounded-loaded wild-type: 17, unloaded wild-type: 14, grounded-loaded tg: 11, unloaded tg: 9. **,## *p* < 0.01 by Tukey–Kramer test * and Student’s *t*-test #. Data were collected from two independent experiments.

To investigate whether the Sp7 tg mice with a sparse and markedly disorganized lacunocanalicular network respond to unloading, we performed tail suspension using wild-type and Sp7 tg mice and compared the unloaded mice with the grounded-loaded mice. After tail suspension for two weeks, the femurs were analyzed by micro-CT and bone histomorphometric analyses (Figure 6, Figure 7 and Figure 8). Based on the micro-CT analysis, the unloaded Sp7 tg and wild-type mice had lower trabecular bone volume, trabecular thickness, trabecular number, and trabecular BMD than the respective value in the grounded-loaded mice (Figure 6A–E). Unloading reduced the cortical bone ratio, cortical thickness, and cortical BMD in the Sp7 tg mice, while it reduced the cortical thickness in the wild-type mice, compared with the respective value in the grounded-loaded mice (Figure 6F–J). The periosteal and endosteal circumferences were unaffected by unloading in both the wild-type and Sp7 tg mice (Figure 6F–J).

We also compared the unloaded mice with the grounded-loaded mice by bone histomorphometric analysis of trabecular bone of the femurs. The osteoblast parameters, including osteoid surface, osteoblast surface, and osteoblast number, were increased, and the osteoclast parameters, including osteoclast surface, osteoclast number, and eroded surface, were increased by unloading in the wild-type mice compared with the grounded-loaded mice (Figure 7). In contrast, these osteoblast and osteoclast parameters were unchanged by unloading in the Sp7 tg mice. Further, the mineralizing surface and bone formation rate were reduced in both the wild-type and Sp7 tg mice by unloading compared with the grounded-loaded mice (Figure 7). The osteoid thickness, osteocyte number, and mineral apposition rate were unaffected by unloading in both the wild-type and Sp7 tg mice (Figure 7).

According to the dynamic bone histomorphometric analysis of cortical bone of femurs, the mineralizing surface and bone formation rate but not the mineral apposition rate in the periosteum and endosteum were reduced by unloading in both the wild-type and Sp7 tg mice compared with the respective value in the grounded-loaded mice (Figure 8).

Thus, bone formation was reduced in both the wild-type and Sp7 tg mice by unloading, but osteoblast and osteoclast parameters in the trabecular bone increased in the wild-type mice, but not in the Sp7 tg mice, after unloading for two weeks.

**Figure 9:**
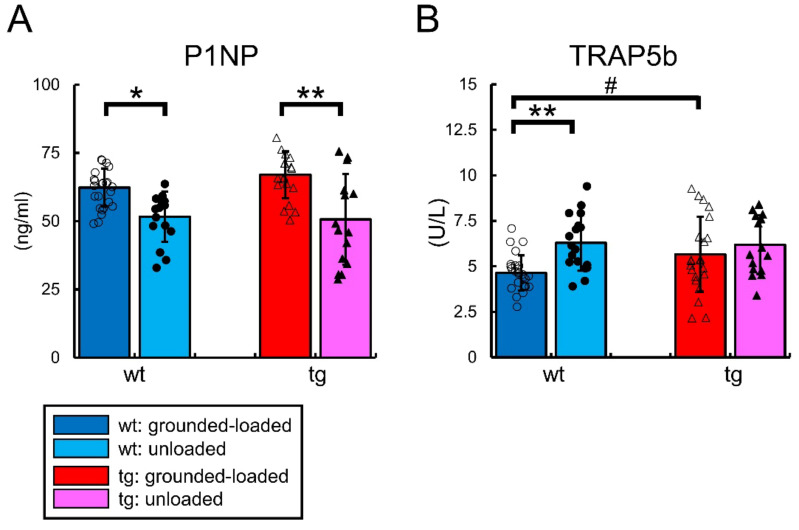
Serum levels of P1NP and TRAP5b in the grounded-loaded and unloaded male mice: (**A**) Total P1NP. (**B**) TRAP5b. The number of mice that were analyzed; grounded-loaded wild-type: 27, unloaded wild-type: 18, grounded-loaded tg: 22, unloaded tg: 15. *^,^# *p* < 0.05, ** *p* < 0.01 by Tukey-Kramer test * and Student’s *t*-test #. The data were collected from two independent experiments.

The serum levels of total P1NP were reduced in both the wild-type and Sp7 tg mice by unloading (Figure 9A). The serum levels of TRAP5b were only increased in the wild-type mice by unloading compared with the grounded-loaded mice (Figure 9B).

### 2.8. The Expression of Osteoblast Marker Genes, Rankl, and Opg in the Osteoblast-Enriched Fraction during Unloading

**Figure 10:**
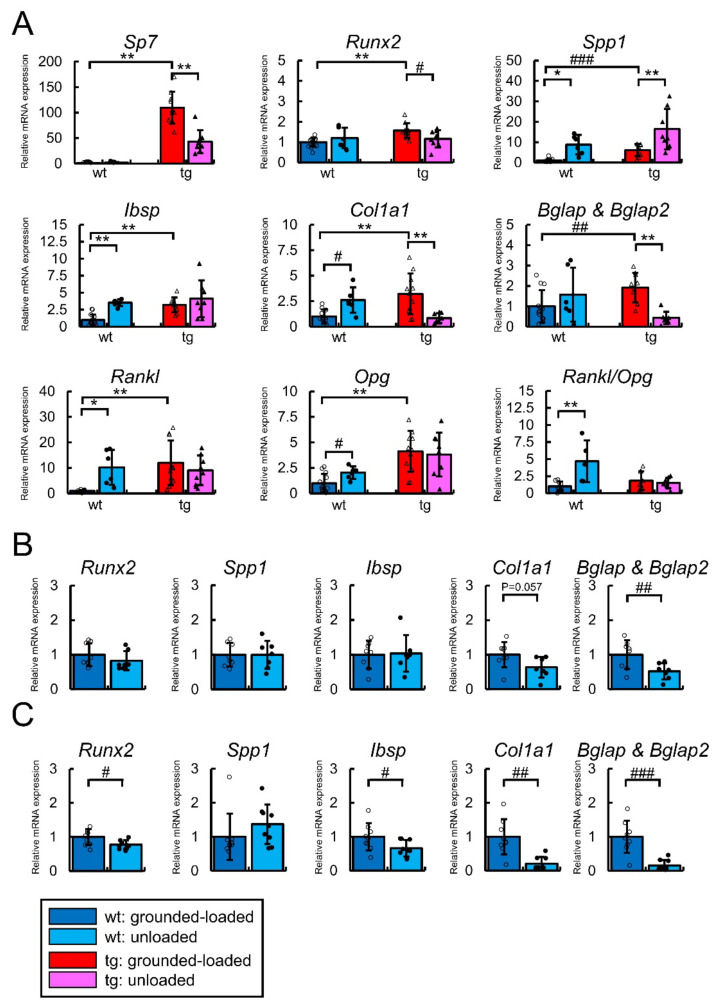
Real-time RT-PCR analyses: (**A**) Tail suspension was performed for two weeks using male wild-type and Sp7 tg mice. The expression of Sp7, Runx2, Spp1, Ibsp, Col1a1, Bglap & Bglap2, Rankl, and Opg, and the Rankl/Opg ratio. The number of mice that were analyzed; grounded-loaded wild-type: 14, unloaded wild-type: 6, grounded-loaded tg: 11, unloaded tg: 9. (**B**) Tail suspension was performed for three days using male C57BL/6 mice. The number of mice that were analyzed; grounded-loaded wild-type: 8, unloaded wild-type: 7. (**C**) Tail suspension was performed for one week using male C57BL/6 mice. The number of mice that were analyzed; grounded-loaded wild-type: 9; unloaded wild-type: 9. In (A–C), the values in the grounded-loaded wild-type mice were defined as one, and relative levels are shown. *^,^# *p* < 0.05, **^,^## *p* < 0.01, ### *p* < 0.001 by Tukey–Kramer test * and Student’s *t*-test #.

The osteoblast-enriched fraction was prepared as described in the Materials and Methods after tail suspension for two weeks. In the wild-type mice, Spp1, Ibsp, Col1a1, Rankl, and Opg expression and Rankl/Opg ratio were increased by unloading compared with the grounded-loaded mice (Figure 10A). In the Sp7 tg mice, the expression of the Sp7 transgene, Runx2, Col1a1, and Bglap/Bglap2 was reduced and that of Spp1 was increased by unloading compared with the grounded-loaded mice (Figure 10A). Thus, Runx2, Col1a1, and Bglap/Bglap2 expression was reduced in the Sp7 tg mice but not in the wild-type mice by unloading. The reduction of Sp7 transgene expression was likely due to the reduction of Col1a1 expression because the transgene was driven by the 2.3-kb Col1a1 promoter. A reduction in the trabecular bone volume and bone formation rate occurred in the first week of tail suspension for two weeks [26]. The osteoblast marker gene expression was also examined in the wild-type mice after tail suspension for three days and one week (Figure 10B,C). Bglap/Bglap2 expression was significantly and Col1a1 expression was marginally reduced by unloading for three days (Figure 10B), whereas Runx2, Ibsp, Col1a1, and Bglap/Bglap2 expression was significantly reduced by unloading for one week (Figure 10C) compared with the respective expression in the grounded-loaded mice. As we used eight-week-old wild-type mice in tail suspension for one week, however, the younger age may have affected the levels of the reduction.

### 2.9. Lack of Differential Expression of Sost in Compression and Tension Sides of Cortical Bone and Increased Serum Sost in Sp7 Tg Mice by Unloading

**Figure 11:**
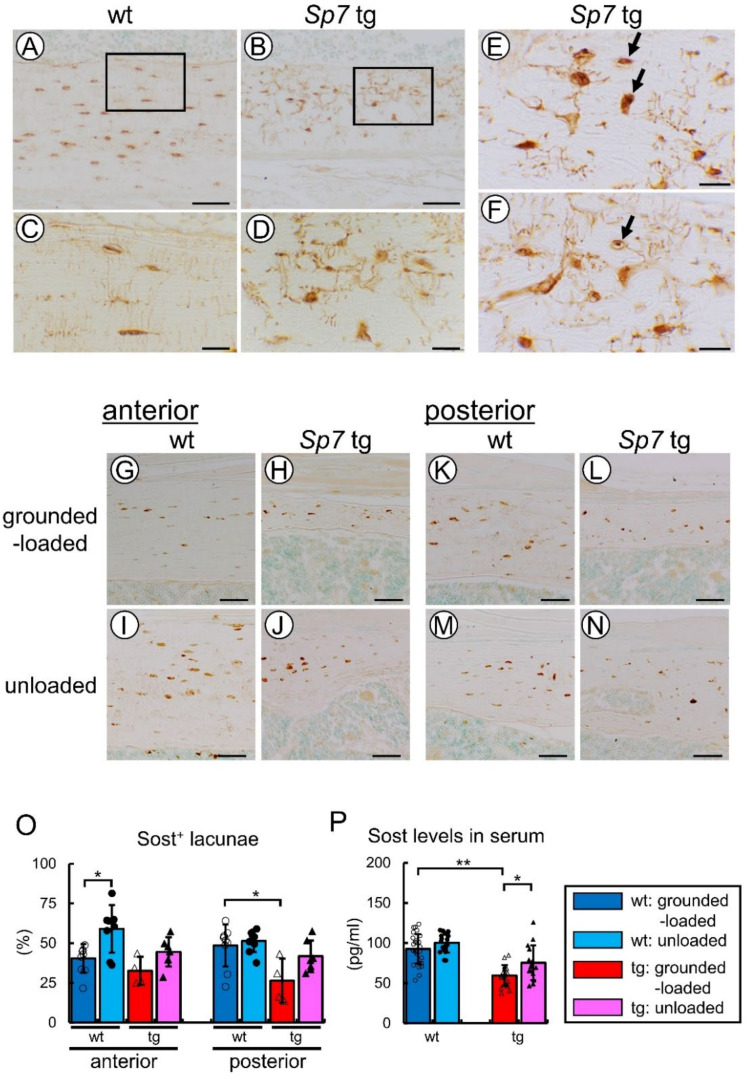
Immunohistochemical analysis of Sost and serum levels of Sost: (**A**–**F**) Immunohistochemistry of the posterior femoral cortical bone in female wild-type (**A**,**C**) and Sp7 tg (**B**,**D**–**F**) mice using anti-Sost antibody. The boxed regions in (**A**,**B**) are magnified in (**C**,**D**), respectively. The arrows in (**E**,**F**) show apoptotic or necrotic osteocytes, which were non-specifically stained with Sost. (**G**-**N**) Immunohistochemistry of the anterior (**G**–**J**) and posterior (**K**–**N**) femoral cortical bone in the grounded-loaded (**G**,**H**,**K**,**L**) and unloaded (**I**,**J**,**M**,**N**) wild-type (**G**,**I**,**K**,**M**) and Sp7 tg (**H**,**J**,**L**,**N**) male mice using anti-Sost antibody. Scale bars = 100 μm (**A**,**B**); 10 μm (**C**–**F**); 50 μm (**G**–**N**). (**O**) Percentages of Sost-positive osteocytes in the anterior and posterior cortical bone. The eight groups were compared. The number of mice that were analyzed; grounded-loaded wild-type: 9, unloaded wild-type: 8, grounded-loaded tg: 4, unloaded tg: 7. * *p* < 0.05. (**P**) Serum levels of Sost in grounded-loaded and unloaded male mice. The number of mice analyzed; grounded-loaded wild-type: 28, unloaded wild-type: 17, grounded-loaded tg: 19, unloaded tg: 15. * *p* < 0.05, ** *p* < 0.01 by Tukey–Kramer test. The data were collected from two independent experiments.

We compared the Sost-positive cells in the anterior (compression side) and posterior (tension side) cortical bone of the grounded-loaded and unloaded mice. After unloading, the percentage of Sost-positive cells increased in the anterior cortical bone, but not in the posterior cortical bone in the wild-type mice, whereas the increase in the percentage of Sost-positive cells was not significant in both the anterior and posterior cortical bone in the Sp7 tg mice compared with the respective percentage in the grounded-loaded mice (Figure 11G–O). The serum Sost level significantly increased in the unloaded Sp7 tg, but not in the unloaded wild-type mice, compared with the respective grounded-loaded group (Figure 11P).

## 3. Discussion

Although the number and function of osteoblasts in the Sp7 tg mice were similar to the wild-type mice, the Sp7 tg mice had thin and porous cortical bone, the osteoclasts and apoptotic osteocytes were increased in the cortical bone, and the Sp7 tg mice exhibited a markedly impaired lacunocanalicular network. However, the Sp7 tg mice responded to unloading by reducing bone formation, at least in part, through the induction of Sost expression, suggesting that the markedly impaired lacunocanalicular network was functionally still enough to respond to unloading. In contrast to the wild-type mice, the Sp7 tg mice lost the ability to differentially-induce Sost by unloading in the compression and tension sides, and failed to restore bone formation during unloading, suggesting that an organized lacunocanalicular network may be required for the regulation of Sost expression and bone formation during unloading. We also revealed that the lacunocanalicular structures differ in the compression and tension sides, in the periosteal and endosteal sides, and in the genders in the wild-type femoral cortical bone, and that the periosteal side in the compression side of the male cortical bone has the most dense and organized lacunocanalicular structure.

The lacunocanalicular network is required for nutrient transport, mechanosensing, and mechanotransduction [27]. As osteocyte apoptosis increased in the cortical bone of the Sp7 tg mice, the lacunocanalicular network may have been insufficient to maintain the osteocyte viability. Osteocyte apoptosis or necrosis further impaired the lacunocanalicular network, because both the intracellular and extracellular communication systems were interrupted by the apoptosis or necrosis of osteocytes (Figure 11E,F), as previously shown in Bcl2 tg mice [12]. However, the Sp7 tg mice responded to unloading, suggesting that the markedly impaired lacunocanalicular network retained a certain capacity for mechanosensing and mechanotransduction. In contrast, we previously reported that the Bcl2 tg mice under the control of the 2.3-kb Col1a1 promoter lost the ability to respond to unloading because a marked reduction of osteocyte processes caused massive osteocyte apoptosis (70% of osteocytes) and led to the complete disruption of the lacunocanalicular network [12,28]. Of note, massive osteocyte apoptosis did not promote bone resorption in Bcl2 tg mice because damage-associated molecular patterns (DAMPs), which are released from necrotic osteocytes that are derived from apoptotic osteocytes and induce osteoclastogenesis, were unable to reach the bone surface and apoptotic osteocytes were unlikely to induce Rankl in the neighboring osteocytes due to the completely disrupted lacunocanalicular network [12,28,29]. Importantly, immunostaining demonstrated that Sost was secreted to the bone marrow and the periosteum irrespective of the markedly impaired lacunocanalicular network, and the serum Sost level was increased by unloading in the Sp7 tg mice. Further, the increased osteocyte density in the male Sp7 tg cortical bone may have contributed to mechanosensing and mechanotransduction. In contrast, Sost was unable to reach the bone surface in Bcl2 tg mice, which lost the response to unloading, and the Sost expression was not changed by unloading [12]. These findings suggest that the lacunocanalicular network in the Sp7 tg mice was markedly impaired but still sufficient for Sost regulation and secretion in unloading.

Collagen fibers run in the longitudinal direction of long bone and the c-axis orientation of apatite crystals is parallel to the collagen fibers to acquire maximum strength in the loading direction [30]. Osteocytes with lacunae align along the bone long axis (loaded direction) with a spindle shape and the canaliculi preferentially extend perpendicular to the bone long axis, but osteocytes and lacunae in disordered collagen fibers are rounded and the canaliculi are oriented isotropically [31,32,33]. The organization of collagen fibers is also important for bone strength [34]. In mouse femurs, the anterior cortical bone is the compression side and the posterior cortical bone is the tension side, and the compression side of cortical bone acquires more bone after loading [15]. Interestingly, the number of osteoclasts in the endosteum of cortical bone in the tension side was higher than that in the compression side in the wild-type femurs (Figure 2A,C,G), suggesting that bone resorption is differentially regulated in the compression and tension sides. Osteocytes had a spindle shape and were aligned in the longitudinal direction, collagen fibers ran in the longitudinal direction, and the canaliculi were aligned perpendicular to the osteocytes in the anterior cortical bone (compression side). In contrast, the osteocytes were rounded in some regions, and the direction of collagen fibers and the alignment of canaliculi were disorganized in the regions especially from the middle to the periosteum of the posterior cortical bone (tension side) in the male wild-type mice (Figure 3 and Figure 4). Further, the canalicular numbers in the periosteal and middle regions in males and in the periosteal region in females were higher in the anterior cortical bone compared with those in the posterior cortical bone in the wild-type mice, and the canalicular numbers in the periosteal and endosteal regions in the anterior cortical bone were higher than those in the posterior cortical bone in the male Sp7 tg mice (Figure 5F–H). Moreover, the lacuna length in the longitudinal direction in the periosteal half of the anterior cortical bone was longer than that of the posterior cortical bone in the male wild-type mice (Figure 5J). Interestingly, the canaliculi in the posterior cortical bone were wider than those in the anterior cortical bone in the wild-type and Sp7 tg mice of both sexes (Figure 5B). These findings suggest that the lacunocanalicular structure in the compression and tension sides is differentially developed, probably by the effect of mechanical stress, and that the compression side in the male wild-type mice is likely to have been most strongly affected by mechanical stress. In accordance with these observations, Sost-positive cells were significantly increased by unloading in the anterior cortical bone (compression side) of the femurs in the male wild-type mice (Figure 11O). It is consistent to our previous report that Sost expression was induced in the compression side of the tibiae of male wild-type mice by unloading [12]. In contrast, the shape of the osteocytes and lacunae was round, the orientation of the collagen fibers and canaliculi was highly disorganized, the canalicular number was markedly reduced, and the increase in the Sost-positive cells by unloading was similar but not significant in the anterior and posterior cortical bone in the male Sp7 tg mice. This suggests that the differential regulation of Sost expression in the compression and tension sides by unloading was lost in the Sp7 tg mice. Thus, these findings suggest that an organized lacunocanalicular network may be required for the differential induction of Sost by unloading in the compression and tension sides of cortical bone.

The woven bone-like structure and the rounded morphology of the osteocytes and lacunae, which were observed in the Sp7 tg mice, are features of immature bone [35,36,37]. It suggests that the osteocytes of the Sp7 tg mice were immature. This may explain why the Sost-positive cells in the posterior cortical bone and the serum level of Sost in the Sp7 tg mice were reduced compared with those in the wild-type mice (Figure 11O,P). It also indicates that osteocyte function was altered in the Sp7 tg mice, and it should have exerted some effects on the response to unloading. In contrast, the trabecular bone volume, bone formation rates in the trabecular and cortical bone, and the serum P1NP were similar between the wild-type and Sp7 tg mice (Figure 1J, Figure 7, Figure 8E and Figure 9A). We previously reported that the expression of osteoblast marker genes, including Col1a1, Ibsp, and Bglap/Bglap2, was lower in Sp7 tg mice than that in wild-type mice at four weeks of age in mixed genetic background of C57BL/6 and C3H [22]. In contrast, the expression of osteoblast marker genes, including Runx2, Spp1, Ibsp, Col1a1, and Bglap/Bglap2, in Sp7 tg mice was higher than that in the wild-type mice at 14 weeks of age in C57BL/6 background (Figure 10A). These findings indicate that osteoblast functions were not impaired in Sp7 tg mice with C57BL/6 background at 14 weeks of age. However, it is still possible that the overexpression of Sp7 exerted some effects on the response to unloading by altering osteoblast functions because Sp7 expression in Sp7 tg mice was reduced by unloading but still much higher than that in the grounded-loaded and unloaded wild-type mice.

As the number of TRAP-positive cells in cortical bone, the osteoclast parameters in trabecular bone, and serum TRAP5b were higher in the Sp7 tg mice than those in the wild-type mice, bone resorption was enhanced in the Sp7 tg mice compared with the wild-type mice (Figure 2G, Figure 7 and Figure 9B). One of the causes for the enhanced bone resorption may be the increased osteocyte apoptosis, and the enhanced bone resorption was likely to have contributed to the formation of porous cortical bone. By unloading, bone resorption was promoted in the wild-type mice, but not in the Sp7 tg mice (Figure 7 and Figure 9B). Consistent with the previous report, unloading increased Rankl expression in the osteoblasts in the wild-type mice [12], but not in the Sp7 tg mice (Figure 10A). As Rankl expression in the Sp7 tg mice was higher than that in the wild-type mice in the grounded-loaded groups, osteocyte apoptosis may have strongly stimulated Rankl expression and unloading may have failed to further stimulate Rankl expression in Sp7 tg mice.

The expression of Runx2, Col1a1, and Bglap/Bglap2 was reduced in the Sp7 tg mice, whereas Col1a1 expression was increased and Runx2 and Bglap/Bglap2 expression was unaffected in the wild-type mice by unloading for two weeks. Further, Bglap/Bglap2 expression was reduced by unloading for three days, and Runx2, Col1a1, and Bglap/Bglap2 expression was reduced by unloading for one week in the wild-type mice. Although these data were obtained only in the wild-type mice due to technical problems, it is conceivable that the expression of these genes was likely to have recovered in the wild-type mice but not in the Sp7 tg mice during unloading for two weeks. As the expression of Spp1, which is expressed in immature osteoblasts [38], was upregulated by unloading in both the wild-type and Sp7 tg mice, Spp1 may be an early marker in the recovery of bone formation. Bone histomorphometric analysis also confirmed the increases in osteoblast parameters only in the wild-type mice after unloading for two weeks. Although bone formation that was based on bone histomorphometric analysis and serum P1NP was reduced in the wild-type mice by unloading, bone formation is likely to have recovered to the level of the grounded-loaded group of wild-type mice at the end of tail suspension for two weeks. Although Spp1 expression was upregulated, a marked reduction of Col1a1 and Bglap/Bglap2 in the Sp7 tg mice at the end of tail suspension for two weeks suggests that an organized lacunocanalicular network may be required for the recovery of the reduced bone formation during unloading. Indeed, altered osteoblast functions in the Sp7 tg mice should have some effects on the recovery.

In summary, we revealed that the lacunocanalicular structure differs in the compression and tension sides, in the periosteal and endosteal sides, and in the genders in wild-type femoral cortical bone, suggesting that the lacunocanalicular structure is developed under the effects of mechanical stress. The Sp7 tg mice were not an ideal model for examining the role of an organized lacunocanalicular structure in mechanoresponse, because the differentiation and functions of osteoblasts should have been affected. However, it is impossible to have an animal model, in which only the lacunocanalicular structure is impaired but the others are normal. Thus, the effects of the impaired lacunocanalicular structure on mechanoresponse must be deduced from the whole phenotypes of the animals with it in the grounded-loaded and loaded/unloaded conditions. From the whole phenotypes of the Sp7 tg mice in the grounded-loaded and unloaded conditions, it was deduced that the sparse and disorganized but connected lacunocanalicular network in the Sp7 tg mice satisfies the minimal requirement for mechanoresponse but a dense and organized lacunocanalicular network is required for the regulation of Sost expression and bone formation in unloaded conditions.

## 4. Materials and Methods

### 4.1. Animal Study

Sp7 tg mice under the control of the 2.3-kb Col1a1 promoter were generated as previously described [22]. The Sp7 tg mice were backcrossed with C57BL/6N mice 8 times before the experiments. The Sp7 tg mice and their littermates were compared at 14 weeks of age. Further, the male Sp7 tg mice and male wild-type littermates were divided into the grounded-loaded and unloaded (tail-suspended) groups at 12 weeks of age. As Sp7 tg mice were fragile, the anesthetized mice were examined by X-ray (micro-FX1000, Fuji film, Inc., Tokyo, Japan) before the tail suspension experiment, and the mice without a fracture were used. Tail suspension was performed for 2 weeks as previously described [12]. The tail suspension was also performed for 3 days using C57BL/6N mice at 14 weeks of age and for one week using C57BL/6N mice at 8 weeks of age. Female Sp7 tg mice and female wild-type littermates were analyzed histologically at 14 weeks of age. Prior to the study, all the experiments were reviewed and approved by the Animal Care and Use Committee of Nagasaki University Graduate School of Biomedical Sciences (No. 1902281515). The animals were housed 3 per cage in a pathogen-free environment on a 12-h light cycle at 22 ± 2 °C with standard chow (CLEA Japan, Tokyo, Japan) and had free access to tap water.

### 4.2. Micro-CT Analysis

Ethanol fixed femurs at 14 weeks of age were analyzed to obtain quantitative data of the trabecular and cortical bone by a micro CT system (R_mCT; Rigaku Corporation, Tokyo, Japan) at a resolution of 10 μm/voxel as previously described [12]. The diaphyseal part of the femurs with a thickness of 2 mm was prepared and the structure of cortical bone was analyzed by Skyscan 1272 (Bruker, Billerica, MA, USA) at a resolution of 0.5 μm/voxel.

### 4.3. Histological Analyses

For the histological analyses, the anesthetized female mice at 14 weeks of age were fixed with 4% paraformaldehyde/0.01 M phosphate-buffered saline (PFA) by perfusion fixation, and the femurs were dissected. The male mice at 14 weeks of age were sacrificed without prefusion fixation, the femurs were dissected, most of the femurs were fixed with ethanol for micro-CT and bone histomorphometric analyses, and some of them were fixed with 4% PFA by immersion fixation. The PFA-fixed femurs were decalcified in 10% EDTA (pH 7.4) and embedded in paraffin. The sections (3 μm thick) were stained with H-E, for TRAP activity, or subjected to immunohistochemistry using anti-Sost antibody (R&D, Minneapolis, MN). TUNEL staining was performed using the ApopTag Peroxidase In Situ Apoptosis Detection Kit S7100 (Merck, Darmstadt, BRD). Bone canalicular staining (silver impregnation staining) was performed using Silver Protein (198-18101: FUJIFILM-Wako) according to the method that was previously described [36]. TRAP staining, TUNEL staining, and Sost immunostaining were analyzed using a Zeiss Axioskop 2 plus microscope (Carl Zeiss, Tokyo, Japan) and an Olympus DP74 camera (Olympus Co., Tokyo, Japan). To examine the direction of the collagen fibers, H-E stained sections were observed in 0.4 mm length in the diaphysis of anterior and posterior cortical bone under polarized light. The TRAP-positive cells were counted in 8 mm length in anterior and posterior cortical bone, the TUNEL-positive cells were counted in 2 mm length in the diaphysis of anterior and posterior cortical bone, and the Sost-positive cells were counted in 0.56 mm length in the diaphysis of anterior and posterior cortical bone. In the analyses of canalicular number, canalicular thickness, and the size and area of lacunae, the sections of canalicular staining were analyzed in 0.55-mm × 0.337-mm area in the diaphysis of the anterior and posterior cortical bone using an All-in-one Fluorescence Microscope (BZ-X700, KEYENCE, Osaka, Japan) with Z-stacks and panorama. A total of 20 pictures of 1000 magnifications were connected to get the 0.55-mm × 0.337-mm area analyzed. In the analysis of canalicular number in the anterior and posterior cortical bone, 10–15 lacunae with live osteocytes were randomly selected, the number of canaliculi in each osteocyte was counted, and the counts were averaged. The anterior and posterior cortical bone was divided into three regions, one-fourth area of the periosteum side, half area in the middle, and one-fourth area of the endosteum side at the mid-diaphysis, and the two longitudinal lines were drawn in the combined pictures of 0.55-mm × 0.337-mm area to divide the cortical bone into the three regions. A total of 5–10 lacunae with live osteocytes were randomly selected in each region, the number of canaliculi in each osteocyte was counted, and the counts were averaged. In the measurement of the canalicular thickness, the width of the randomly selected 20 canaliculi around lacunae with live osteocytes was measured and averaged. In the measurement of the size and area of lacunae, the anterior and posterior cortical bone was divided into the periosteal and endosteal halves at the mid-diaphysis, and a longitudinal line was drawn in the combined pictures of 0.55-mm × 0.337-mm area to divide the cortical bone into the two regions. A total of 10 lacunae with live osteocytes were randomly selected in each region, the longitudinal and vertical lengths and area of each lacuna were measured, and the values were averaged. In each experiment, one section was analyzed in each mouse. The ethanol-fixed undecalcified femurs were embedded in glycolmethacrylate for bone histomorphometric analysis. For assessment of the dynamic histomorphometric indices, the mice were injected with calcein at 10 d and 2 d before sacrifice at a dose of 0.16 mg/10 g body weight. Bone histomorphometric analyses were performed as previously described [21].

### 4.4. Serum Testing

The serum levels of total P1NP, TRAP5b, and Sost were measured using rat/mouse P1NP ELISA (Immunodiagnostic Systems, Boldon, UK), Mouse TRAP Assay (Immunodiagnostic Systems), and mouse/rat SOST/Sclerostin Quantikine ELISA Kit (R&D Systems, Minneapolis, MN, USA), respectively.

### 4.5. Real-Time RT-PCR

Muscle, connective tissue, and periosteum were removed from the tibiae of the control and unloaded mice at 14 weeks of age and C57BL/6 mice at 9 and 14 weeks of age, and the bones were cut at the metaphyses. After the hematopoietic cells in the diaphyses of tibiae were flushed out with PBS, the osteoblast-enriched cells were collected using a microintertooth brush (Kobayashi Pharmaceutical Co. Ltd., Osaka, Japan) as described previously [12]. The osteoblast-enriched cells were lysed and homogenized using ISOGEN (Wako, Osaka, Japan), RNA-containing aqueous layer was separated by chloroform, and the RNA was precipitated by isopropanol. A total of 0.5 μg RNA was reverse transcribed using ReverTra Ace^®^ qPCR RT Master Mix with gDNA Remover (Toyobo, Osaka, Japan). Real-time RT-PCR was performed using THUNDERBIRD SYBR qualitative PCR (qPCR) Mix (Toyobo) and a Light Cycler 480 (Roche Diagnostics, Tokyo, Japan) using the following primers: actin beta (Actb), 5′-CCACCCGCGAGCACAGCTTC-3′/5′-TTGTCGACGACCAGCGCAGC-3′; Sp7 transcription factor (Sp7), 5′-AGGCACAAAGAAGCCATAC-3′/5′-AATGAGTGAGGGAAGGGT-3′; runt related transcription factor 2 (Runx2), 5′-AACAAGACCCTGCCCGTG-3′/5′-TGAAACTCTTGCCTCGTCCG-3′; secreted phosphoprotein 1 (Spp1), 5′-GCAGAATCTCCTTGCGCCAC-3′/5′-CGAGTCCACAGAATCCTCGC-3′; integrin binding sialoprotein (Ibsp), 5′-TGGAGACGGCGATAGTTC-3′/5′-CTAGCTGTTACACCCGAGAG-3′; and bone gamma carboxyglutamate protein and bone gamma-carboxyglutamate protein 2 (Bglap & Bglap2), 5′-ACTCCGGCGCTACCTTGGAGCC-3′/5′-GCAGGGTTAAGCTCACACTG-3′, and the following TaqMan probes: collagen type I alpha 1 (Col1a1), Thermo Fisher (Mm00801666_g1); receptor activator of NF-κB ligand (Rankl), Thermo Fisher (Mm00441906_m1); osteoprotegerin (Opg), Thermo Fisher (Mm1205928_m1). We normalized the values to those of Actb.

### 4.6. Statistical Analysis

The values are shown as the mean ± SD. Statistical analyses were performed by the Student’s t-test using BellCurve for Excel (Social Survey Research Information Co., Ltd., Tokyo, Japan) in the comparison of two groups and by the Tukey–Kramer test in the comparison of more than four groups. A *p*-value < 0.05 was considered significant. A significant difference by the Student’s *t*-test was shown with # and that by the Tukey–Kramer test was shown with *.

## Data Availability

No available data.
